# The Effect of Coenzyme Q10 on Liver Injury Induced by Valproic Acid and Its Antiepileptic Activity in Rats

**DOI:** 10.3390/biomedicines10010168

**Published:** 2022-01-13

**Authors:** Fahad Alqarni, Hala S. Eweis, Ahmed Ali, Aziza Alrafiah, Mohammed Alsieni, Shahid Karim, Mosleh Ayed Alkathyri

**Affiliations:** 1 Department of Pharmacology, Faculty of Medicine, King Abdulaziz University, Jeddah 21589, Saudi Arabia; ph.fahadalgarni@gmail.com (F.A.); heweis@kau.edu.sa (H.S.E.); profahmedali@gmail.com (A.A.); malsieni@kau.edu.sa (M.A.); shahid.karim@yahoo.co.in (S.K.); 2 Department of Medical Laboratory Sciences, Faculty of Applied Medical Sciences, King Abdulaziz University, Jeddah 21589, Saudi Arabia; 3 Pharmaceutical Care Administration, Directorate of Health Affairs, Ministry of Health, Taif 26315, Saudi Arabia; malkatheri@moh.gov.sa

**Keywords:** pentylenetetrazole, valproic acid, coenzyme Q10, glutathione, malondialdehyde, Protein Carbonyl, reactive oxygen species

## Abstract

Valproic acid (VPA) has toxic metabolites that can elevate oxidative stress markers, and the hepatotoxicity of VPA has been reported. Coenzyme Q10 (CoQ10) is one of the most widely used antioxidants. The effect of CoQ10 on epileptogenesis and VPA hepatotoxicity were examined. Rats were randomly divided into five groups: the control group received 0.5% methylcellulose by oral gavages daily and saline by intraperitoneal injection three times weekly. The PTZ group received 1% methylcellulose by gavages daily and 30 mg/kg PTZ by intraperitoneal injection three times weekly. The valproic acid group received 500 mg/kg valproic acid by gavage and 30 mg/kg PTZ, as above. The CoQ10 group received 200 mg/kg CoQ10 by gavages daily and 30 mg/kg PTZ, as above. The Valproic acid + CoQ10 group received valproic acid and CoQ10, as above. Results: CoQ10 exhibited anticonvulsant activity and potentiated the anticonvulsant effect of VPA. CoQ10 combined with VPA induced a more significant reduction in oxidative stress and improved the histopathological changes in the brain and liver compared to VPA treatment. In addition, CoQ10 reduced the level of toxic VPA metabolites. These findings suggest that the co-administration of CoQ10 with VPA in epilepsy might have therapeutic potential by increasing antiepileptic activity and reducing the hepatotoxicity of VPA.

## 1. Introduction

Oxidative damage can affect neuron excitability with increased susceptibility to seizure attacks. Furthermore, oxidative damage and mitochondrial dysfunction with excessive free radical production in the organs, in addition to the imbalance between free radical concentrations and antioxidant defenses, may not only be associated with epileptogenesis pathology but also has been linked to the pathogenesis of liver fibrosis, stroke, diabetes mellitus, and atherosclerosis [1].

However, regardless of the variety of anti-epileptic drugs (AEDs), there still has not been a complete improvement of epilepsy. More than 30% of patients have drug-resistant epilepsy, in addition to other limitations in some AED treatments [2,3]. Furthermore, some AEDs have toxic metabolites that can elevate oxidative stress and mitochondrial dysfunction markers in other organs, such as liver toxicity associated with chronic valproic acid (VPA) use; VPA metabolites cause oxidative stress as a consequence of elevated ROS creation [4]. These limitations of conventional AEDs highlight the necessity of the investigation of additional treatments that can potentiate traditional anti-epileptics and prevent their adverse effects.

Valproic acid is one of the broadest-spectrum AEDs, and is generally regarded as a first-choice agent for newly diagnosed epilepsy [5]. However, hepatic toxicity has been linked to the chronic use of the drug. In addition, oxidative stress related to excessive ROS production and weak antioxidant capability has been theorized to contribute to hepatic toxicity [4,6]; this severe toxic effect justifies the addition of a hepatoprotective drug to minimize, inhibit, or reverse the hepatotoxicity induced by VPA. Therefore, the search for novel AEDs with antioxidant properties and the investigation of their antiepileptic activity is warranted.

Antioxidants have become popular in contemporary research because they can neutralize free radicals [7] and have the potential to become therapeutic agents to help counteract ROS-mediated damage to neurons [8]. This aspect has been captured in current research efforts that seek to explore the extent to which antioxidants can help alleviate factors that might exacerbate neuronal epilepsy and the side effects of AEDs. Coenzyme Q10 (CoQ10) is one of the most widely used antioxidants [9]. Accordingly, the present study was conducted to examine the effect of CoQ10 in the epileptogenesis process—either alone or combined with valproic acid—using a rat model of pentylenetetrazole (PTZ)-induced kindling, in addition to the evaluation of the effect of CoQ10 against valproate-induced hepatotoxicity.

## 2. Materials and Methods

### 2.1. Tested Drugs

The VPA sodium (Sigma-Aldrich Chemicals, Saint Louis, MI, USA) was supplied as a white, odorless powder dissolved in distilled water to a standard 50 mg/mL solution for oral administration. The CoQ10 (NOW, Chicago, MA, USA) was supplied as a yellow, odorless powder suspended in distilled water + methylcellulose 1%. The PTZ (Metrazole Sigma-Aldrich Chemicals, St. Louis, MO, USA) was supplied as a white crystalline powder dissolved in saline, at 0.9% NaCl, to a standard 25 mg/mL concentration solution for intraperitoneal injection.

### 2.2. Methods

#### 2.2.1. Study Design

##### Animals

Fifty male Wistar rats, aged 6–8 weeks old with 180–250 g body weight, were purchased from the animal house at the King Fahad Medical Research Center, Department of Pharmacology, Faculty of Medicine, King Abdulaziz University, Jeddah, Saudi Arabia. The rats were housed in groups of 10, divided into a batch of 5 rats each in standard clear polycarbonate cages (360 cm × 180 cm × 220 cm) with free access to food and water. The animals were kept on a 12-h light–dark cycle daily, in a controlled environment, at a temperature of 25 °C, and they received good care in compliance with ethical standards. All of the experiments were conducted during the light phase, from 10:00 to 16:00. The experimental protocol was approved by the Institutional Animal Care and Use Committee (ACUC) and Research Ethics Committee (REC), with reference number (596-17).

##### Animal Groups

The rats were randomly divided into five groups, each with ten rats, as follows: The control group received 1% methylcellulose by oral gavages daily, and saline by intraperitoneal injection three times weekly. The PTZ group received 1% methylcellulose by gavages daily and 30 mg/kg PTZ by intraperitoneal injection three times weekly. The valproic acid group received 500 mg/kg of valproic acid by gavage and 30 mg/kg PTZ by intraperitoneal injection three times weekly. The CoQ10 group received 200 mg/kg CoQ10 by gavages daily and 30 mg/kg PTZ by intraperitoneal injection three times weekly. The valproic acid + CoQ10 group received 500 mg/kg of valproic acid, 200 mg/kg CoQ10 by gavage daily, and 30 mg/kg PTZ by intraperitoneal injection three times weekly.

The valproic acid dose used was 500 mg/kg, and was selected based on El-Mowafy et al. [10]. The PTZ dose used in the present study (30 mg/kg) was selected based on Attack et al. [11]. The coenzyme Q10 dose used (200 mg/kg) was selected based on Matthews et al. [12]. All of the oral tested drugs were suspended in 1% methylcellulose, and were freshly prepared before each test [13]. The experiment was started by the receipt of oral tested drugs, and the next day, the mice received a PTZ injection one hour after the administration of the oral drugs. The duration of the experiment was up to 8 weeks. The rats showed full kindling, which was defined as exhibiting a stage 4 or 5 seizure score on three consecutive trials [14]. All of the rats were weighed weekly, and the doses were adjusted accordingly.

#### 2.2.2. Experimental Procedure

##### Assessment of the Epileptogenic Effect Induced by PTZ Kindling

The rats were injected with a sub-convulsive dose of PTZ (30 mg/kg) on every alternate day for eight weeks (chemical kindling), either alone in the model group (PTZ group) or preceded by the oral administration of the test drugs in the drug-treated groups for eight weeks [15]. The rats were individually placed in a plexiglass chamber (10” × 18” × 18”) for behavioral observations over 30 min. Behavioral changes such as seizures, head nodding, jerking movements, and increased urination and defecation were observed, along with the mortality rate.

1. Racine score: The rats were observed for 30 min after the administration of the sub-convulsant dose of PTZ, and the seizure activity was scored using a scoring system from 0 to 5 (Table 1):
2. Development of PTZ kindling (GTCS) in days: The rats were considered kindled if GTCS occurred on two consecutive injections.
3. Latency to GTCS: The onset of GTCS immediately following PTZ administration was recorded in seconds at the end of the 8th week.
4. Duration of GTCS: Immediately following the PTZ administration, the duration of GTCS was recorded in seconds at the end of the 8th week.

##### Behavioral Assessment by the Rota Rod Performance Test

The Rota Rod machine evaluated all of the rats for motor coordination (locomotors). It measured balance, coordination, and motor control. The effects of the substances on motor coordination were measured the next day, after the last PTZ injection.

Procedure:

1. Before testing: In a day free from PTZ injection, threats were placed in the testing room for at least 1 h for habituation and training. Each rat should be able to walk forward on a rotating rod at (12 rpm) for at least 60 s, repeated for a total of three trials separated by 5 min inter-trial intervals.
2. Testing procedure: After the last PTZ injection, each rat was placed on the rotating rod (7 cm diameter) in separate lanes, at a speed of 12 rpm. The time spent on the rod was measured up to 180 s (latency to fall) [16].

#### 2.2.3. Biochemical Studies

##### Sample Collection

At the end of the behavioral assessment, the next day after the last PTZ injection, the rats were anesthetized by 1.2 gm/Kg urethane by intraperitoneal injection. Then, a blood sample was collected from the ophthalmic venous plexus through a retro-orbital approach, and the animals were decapitated to obtain the brain and liver tissues. The liver tissue was weighted and then divided into two parts: one was stored at −80 °C until the biochemical assessment, and the second part was kept in 10% formaldehyde for histological studies. Furthermore, the brain tissue of 5 animals was divided into two parts, and each was kept in the Eppendorf after being weighted, at −80 °C; one for biochemical assessment and the second for high-performance liquid chromatography. Meanwhile the other remaining brain tissues of 2 animals were kept in 10% formaldehyde for the histological studies. Furthermore, the cerebral cortexes of all of the animals were kept in a cryovial and stored at −80 °C.

##### Sample Preparation

- Serum Preparation

The blood sample was collected and then placed in the shaker machine for 30 min, allowing the sample to clot. Then, the sample was centrifuged for 5 min at 3500 rpm, and the supernatant serum was separated by pipette and immediately stored at −20 °C until it was assayed.

- Liver Homogenate Preparation

The liver tissues, kept at −80 °C for biochemical assessment, were rinsed in ice-cold phosphate buffer saline (PBS) at pH = 7.4. Then, the tissues were minced into small pieces and homogenized with PBS at a concentration of 100mg tissue/mL PBS using Brinkman instruments on ice. The resulting suspension was subjected to two freeze–thaw cycles to break the cell membrane further. Next, the suspension was centrifugated for 15 min at 1500× *g* (or 5000 rpm); then, the supernatant was collected and stored at −80 °C until it was assayed.

- Brain Homogenate Preparation

The brain tissues (hippocampus) of all of the groups, kept at −80 °C for biochemical assessment, were rinsed in ice-cold phosphate buffer saline (PBS) at pH = 7.4. Then, the tissues were minced into small pieces and homogenized with PBS at a concentration of 100mg tissue/mL PBS using a tissue lysate machine, and then immediately put on ice. The resulting suspension was subjected to two freeze-thaw cycles to break the cell membrane further. Next, the suspension was centrifugated for 15 min at 1500× *g* (or 5000 rpm); then, the supernatant was collected and stored at −80 °C until it was assayed. At the same time, the other parts of the brain tissues (the hippocampus and cerebral cortex) were kept at −80 °C for HPLC assessment.

##### Serum Liver Function Test (LFE)

The liver enzymes were measured using a Rat Alanine aminotransferase (ALT) ELISA Kit (Catalog number MBS269614) and a Rat Aspartate aminotransferase (AST) ELISA Kit (Catalog Number: MBS264975). All of the procedures were carried out as described in the manufacture manual.

##### Serum Uric Acid Concentration

The uric acid was measured using a uric acid microplate assay kit (UA) (Catalog Number: MBS8243236). All of the procedures were carried out as described in the manufacturer’s manual.

##### Serum Urea Concentration

The urea was measured using a Urea assay kit (Urease method) (Catalog Number: MBS2540560). All of the procedures were carried out as described in the manufacturer’s manual.

##### Antioxidant, Oxidative Stress and Inflammatory Markers in the Hippocampal and Liver Homogenate

The reduced glutathione (GSH) was competitively measured in tissue homogenate using a rat reduced glutathione (RGLU) ELISA kit (Catalog Number: MBS724319). The lipid peroxidation (MDA) was measured using a rat malondialdehyde Elisa kit (Catalog Number: MBS738685). A Protein Carbonyl (PC) ELISA kit (Catalog Number: MBS726854) was used for the quantitative measurement of the PC. A serum tumor necrosis factor-α (TNF-α): PicoKine (TNF-α) ELISA kit (Catalog Number: MBS175904) was used for the quantitative detection of rat TNF-a in the serum. A reactive oxygen species (ROS) assay kit (Catalog Number: MBS2540517) was used to detect the intracellular reactive oxygen species in the tissue homogenate.

##### Cytochrome P-450 2E-1 (CYP2E1) in the Liver Homogenate

Rat cytochrome P450 2E1/(CYP2E1) ELISA kit (Catalog Number: MBS725963) was used to measure the concentration of CYP2E1 in the tissue homogenate using the quantitative sandwich immunoassay technique.

##### Measuring the Concentration of Glutamate in the Hippocampal and Cerebral Cortex Homogenates 

HPLC was used for the quantitative measurement of the glutamate in the cerebral tissue in order to indicate neuro-excitatory activity.

##### Quantification of Glutamate in the Brain

The brain tissues were homogenized in distilled water and heated at 98 °C for 5 min. The samples were then centrifuged at 10,000 rpm for 5 min, at 4 °C. After the centrifugation, the pellets were re-suspended in 1N NaOH in order to determine the total protein amount using the Lowry method (Waterborg and Matthews 1994). In addition, the supernatants were filtered through a 0.22 μ filter, and were then derivatized with sodium sulfite and O-phthalaldehyde; they were then further injected onto a C18 HPLC column (100 × 2.1 MM),(PetraScientific Technologies L.L.C item number# ACE-111-1002), which was inserted into the Shimadzu Prominence UFLC to measure the glutamate concentrations. The glutamate tissue contents were detected using the PDA Photodiode Array Detector. The concentrations in each sample were analyzed according to their peak area compared to the external standard and normalized to protein content.

##### Detection of the Total Protein in the Homogenate

The protein contents in the hippocampal and hepatic homogenates’ supernatants were measured by the Lowry method using bovine serum albumin as the standard in the Bradford protein assay (Catalog number MBS 355526).

#### 2.2.4. Histopathological Studies

##### Hippocampus Histopathological Studies

A. Hippocampal 5 μm-thickness paraffin sections of the brain were stained with Hematoxylin and Eosin (H&E) and Nissl Staining to detect the Nissl granules [17].

B. Hippocampal Immunohistochemical Staining.

The Glial fibrillary acidic protein (GFAP) technique was used to stain the astrocytes [17].

##### Liver Histopathological Studies

The right lobe of the liver was excised. The excised slices of liver were fixed in 10% formaldehyde for one week. This was followed by dehydration, clearing, and embedding in paraffin. Serial sections were cut at a thickness of 5 µm, and were subjected to the following techniques:

A. H&E staining
B. Mallory’s triple stain, which detects collagen fibers that appear blue.

### 2.3. Statistical Analysis

Results are expressed as the mean ± S.E.M. The statistical analysis was performed using one-way analysis of variance (ANOVA) with Tukey’s post hoc test. All of the statistical analyses were performed using the package version SPSS statistical software. A *p* < 0.05 was considered as significant.

## 3. Results

### 3.1. Effects of CoQ10 on the Course of PTZ Kindling in Rats

In contrast to non-PTZ-treated rats (the control negative group), in which no epileptic activity or deaths were documented, the rats subjected to PTZ kindling exhibited epileptic activity and an increased mortality rate. The rats subjected to PTZ kindling exhibited a 30% mortality rate, while those treated with VPA or CoQ10 showed a 20% mortality rate. However, the ASTA/VPA treated group reported no death.

The effects of the test drugs on the course of PTZ kindling are summarized as follows.

#### Effects of CoQ10 on the Racine Score in Rats Subjected to PTZ Kindling

**Figure 1:**
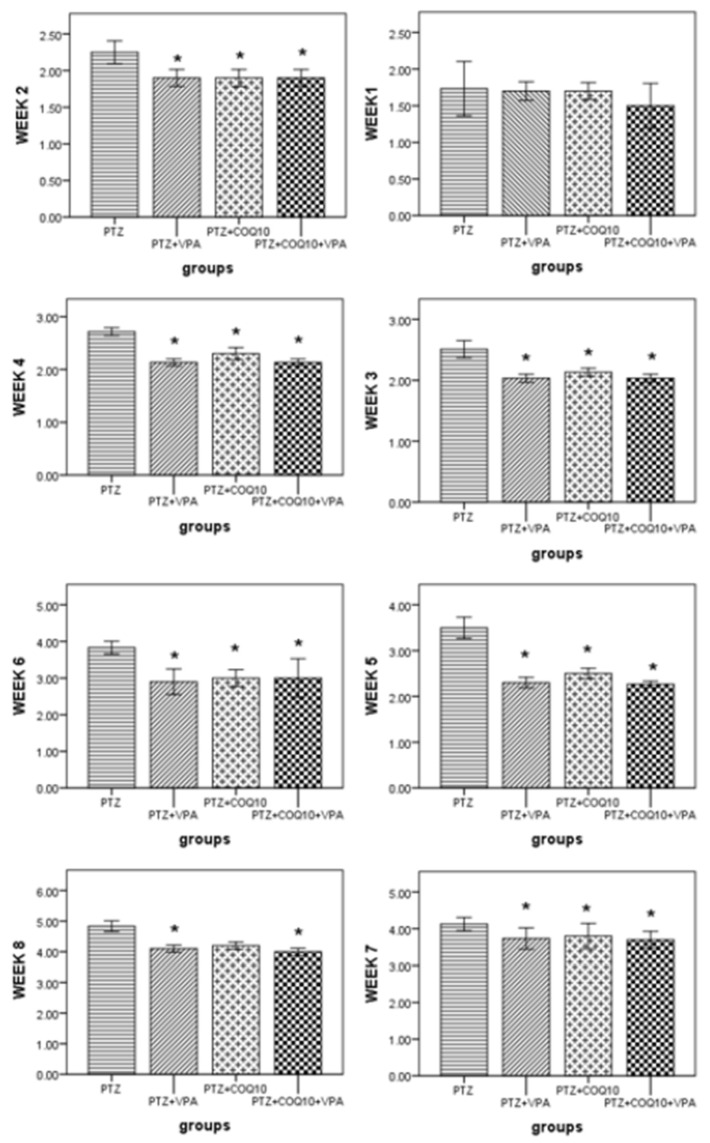
Effects of CoQ10 on the kindling course of Pentylenetetrazole. The data are expressed as the mean ± the standard error of the mean (*N* = 6), one-way ANOVA, and a post hoc Tukey’s test. * *p* < 0.05 compared to PTZ alone group.

**Figure 2:**
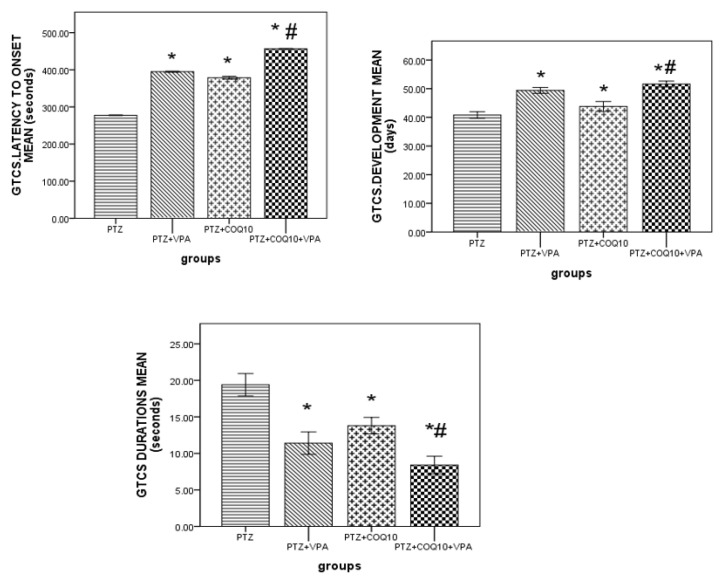
Effects of COQ10 on the kindling development, latency and duration in rats subjected to Pentylenetetrazole. The data are expressed as the mean ± the standard error of the mean (*N* = 6), one-way ANOVA, and post hoc Turkey’s test. * *p* < 0.05 compared to the PTZ alone group; # *p* < 0.05 for the PTZ/VPA + COQ10 group compared to the PTZ/VPA group.

The PTZ-treated rats exhibited a gradual increase in their Racine score, which started at 1.7 ± 0.11 in the first week, and reached 4.8 ± 0.08 by the end of the eighth week. The VPA treatment and PTZ resulted in a significantly (*p* < 0.05) lower Racine Score by the end of the third, fourth, fifth, sixth, seventh, and eighth weeks compared to the PTZ-treated rats. CoQ10 resulted in a significantly (*p* < 0.05) lower Racine Score than that of the rats receiving PTZ alone at the end of the second week, up until the seventh week. The group receiving the combination of both drugs showed a significantly (*p* < 0.05) lower Racine Score than in the PTZ-treated rats by the end of the second week up to the end of the eighth week. The rats treated with VPA and COQ10 demonstrated a significant (*p* < 0.05) delay in GTCS latency, duration and development after the injection of PTZ at the end of week eight (Figure 1). The VPA/COQ10 combination group showed a more significant (*p* < 0.05) delay in GTCS latency, and a decrease in the duration and time of the GTCS development after the injection of PTZ versus the PTZ and PTZ/VPA groups (Figure 2).

### 3.2. Effect of CoQ10 on the Pentylenetetrazole Rats’ Behavior according to the Rota Rod Performance Test

**Figure 3:**
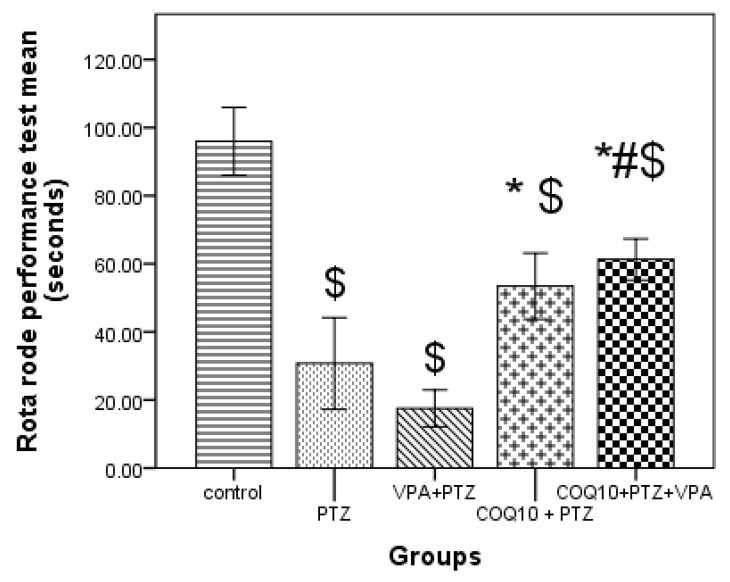
Effects of COQ10 on a Pentylenetetrazole rat’s behavior according to the rota rod performance test. The data are expressed as the mean ± the standard error of the mean (*N* = 6), one-way ANOVA, and post hoc Tukey’s test. $ *p* < 0.05 compared to group I (control); * *p* < 0.05, compared to PTZ alone group; # *p* < 0.05 for the PTZ/VPA + COQ10 group compared to group PTZ/VPA.

- As shown in Figure 3, the PTZ/COQ10 treatment group resulted in a significant (*p* < 0.05) increase in riding time compared to the animals treated with PTZ alone. The VPA/COQ10 combination group showed a significant (*p* < 0.05) increase in riding time compared to the PTZ/VPA-treated rats.

### 3.3. Effects of CoQ10 Test Drugs on Antioxidants and Oxidative Stress Markers

#### 3.3.1. Oxidative Stress Marker and Glutathione in Hippocampus Tissues of PTZ-Treated Rats

As shown in Table 2, PTZ-treated rats had significantly (*p* < 0.05) lower levels of GSH compared to control. In addition, significantly (*p* < 0.05) higher MDA, PC, and ROS levels were noted for PTZ-treated rats than in the control group. VPA treatment during PTZ kindling resulted in significantly higher (*p* < 0.05) levels of GSH compared to PTZ-treated rats. In addition, significantly (*p* < 0.05) lower MDA levels than PTZ treated group and PC were also noted. PTZ/CoQ10 treatment resulted in significantly (*p* < 0.05) lower levels of MDA, PC but not in ROS compared to rats treated with PTZ alone. However, COQ10 has shown a significantly higher level of GSH compared with PTZ alone.

Rats treated with the combination of VPA and COQ10 showed significantly higher levels of GSH compared to the PTZ-treated rats. However, there were significantly (*p* < 0.05) lower MDA, ROS, and PC levels in the PTZ-treated rats. In addition, when comparing the combination group with the PTZ/VPA-treated rats, there were significantly high levels of GSH compared to the PTZ/VPA-treated rats, and significantly lower levels of MDA and ROS compared to the PTZ/VPA-treated rats; however, there was no deferent in PC when compared with the PTZ/VPA-treated rats.

#### 3.3.2. Quantification of Glutamate in the Hippocampus and Cortex Tissue Using a High-Performance Liquid Chromatography Test

**Figure 4:**
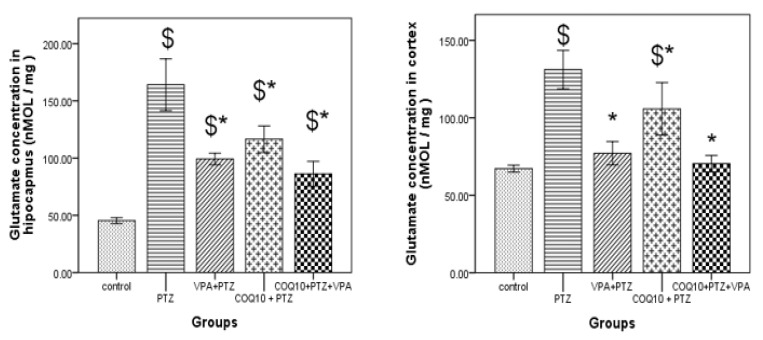
Effect of the test drugs on the glutamate level in the hippocampus and cortex of rats subjected to PTZ kindling. The data are expressed as the mean ± the standard error of the mean (*N* = 6), one-way ANOVA, and post hoc Tukey’s test. COQ10: Coenzyme Q10; PTZ: Pentylenetetrazole; VPA: Valproic acid. $ *p* < 0.05, compared to the control group; * *p* < 0.05, compared to the PTZ group alone.

As shown in Figure 4, the PTZ-treated rats had significantly (*p* < 0.05) higher hippocampus glutamate levels than the control. In addition, significantly (*p <* 0.05) higher levels of cortical glutamate were noted for the PTZ-treated rats than those in the control group. VPA treatment during PTZ kindling resulted in significantly low (*p* < 0.05) levels of hippocampus glutamate for the PTZ/VPA-treated rats compared to the PTZ-treated animals. In addition, significantly (*p* < 0.05) lower cortical glutamate levels than PTZ treated group. PTZ/COQ10 treatment resulted in significantly low (*p* < 0.05) levels of hippocampus glutamate compared to PTZ-treated animals. In addition, there were significantly (*p* < 0.05) lower cortical glutamate levels than those in the PTZ treated group.

The rats treated with the combination of VPA and COQ10 resulted in significantly (*p* < 0.05) lowest levels of hippocampus glutamate compared to the PTZ-treated animals. In addition, they had the significantly (*p* < 0.05) lowest levels of cortical glutamate compared to PTZ treated group.

The VPA and COQ10-treated rats were not significantly different in the glutamate level of the brain hippocampus and cortex compared to the PTZ+VPA-treated rats, but demonstrated the lowest concentration compared with the others.

#### 3.3.3. Effects of the CoQ10 Test Drugs on the Antioxidants and Oxidative Stress Markers in the Hepatic Tissue of the PTZ-Treated Rats

As shown in Table 3, the PTZ-treated rats had significantly (*p* < 0.05) lower levels of GSH compared to the control rats. In addition, significantly (*p* < 0.05) higher levels of MDA, PC, ROS, and Cytochrome P-450 2E1 CYP2EL enzyme were noted for the PTZ-treated rats than the control group. The VPA treatment during the PTZ kindling resulted in significantly higher (*p* < 0.05) levels of GSH compared to the PTZ-treated animals. In addition, there was a significant difference (*p* < 0.05) in the CYP2EL enzyme but not in the MDA and ROS levels compared to the PTZ-treated group.

The PTZ/COQ10 treatment resulted in significantly (*p* < 0.05) lower levels of MDA, ROS, PC, and CYP2EL enzymes compared to the rats treated with PTZ alone; in addition, COQ10 showed a significantly high level of GSH enzymes compared with PTZ alone.

The rats treated with the combination of VPA and COQ10 showed significantly higher levels of GSH compared to the PTZ-treated rats. Significantly (*p* < 0.05) lower MDA, ROS, PC, and CYP2EL enzyme levels were noted.

In addition, when comparing the combination group with the PTZ/VPA-treated rats, significantly high levels of GSH compared to the PTZ/VPA-treated rats were noted, along with significantly lower levels of MDA and ROS, but there was no deferent in PC or CYP2EL enzymes when compared with the PTZ/VPA-treated rats.

#### 3.3.4. Effects of COQ10 on VPA-Induced Liver Toxicity

**Figure 5:**
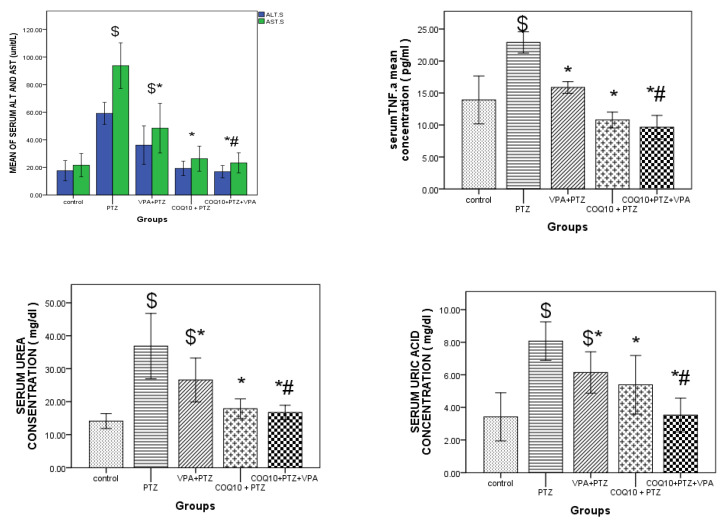
Effect of CoQ10 on liver ALT, AST, and TNF. Pictured are the uric acid and urea of the PTZ/VPA-treated rats. The data are expressed as the mean ± the standard error of the mean, one-way ANOVA, and a post hoc Tukey’s test. ALT: Alanine aminotransferase AST; Aspartate aminotransferase; CoQ10: Coenzyme Q10; PTZ: Pentylenetetrazole; VPA: Valproic acid; TNF.a: tumor necrosis factor. $ *p* < 0.05, compared to group I (control); * *p* < 0.05, compared to the PTZ-alone group; # *p* < 0.05 for the PTZ/VPA + CoQ10 group compared to the PTZ/VPA group.

As shown in Figure 5, VPA treatment during the PTZ kindling resulted in significantly higher (*p* < 0.05) ALT levels and AST compared to the combination of PTZ/VPA and COQ10. In addition, we noted significantly higher (*p* < 0.05) levels of TNFα, urea, and uric acid compared to the combination of PTZ/VPA and COQ10. Conversely, PTZ/COQ10 treatment resulted in significantly (*p* < 0.05) lower ALT levels and AST than PTZ only. In addition, we noted significantly lower (*p* < 0.05) levels of tumor necrosis factor TNFα, urea, and uric acid compared to PTZ only.

##### Hippocampus Histology

**Figure 6:**
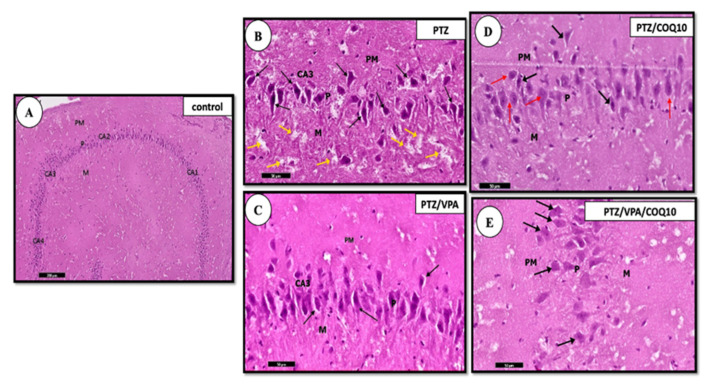
A photomicrograph of a parasagittal section of the hippocampus of all of the rat groups. (**A**) The negative control showing the normal architecture of the hippocampus. (**B**) The PTZ group showing the predominance of the degenerated, deeply stained, shrunken neurons with per neuronal spaces within the pyramidal layer (↑). Notice the pale, vacuolated areas in the molecular layer (M) suggesting the concurrent swelling/degeneration of the pyramidal cell axons (yellow ↑). (**C**) The PTZ/VPA group showing the presence of occasional, deeply darkly stained cells (↑). (**D**) The PTZ/COQ10 group showing most of the pyramidal cells, which have large rounded vesicular nuclei (red ↑). Few deeply stained cells are present with per neuronal spaces (black ↑). (**E**) The PTZ/VPA/COQ10 group showing the pyramidal layer which formed of pyramidal cells (↑) with vesicular nuclei and basophilic cytoplasm. The outer polymorphic layer, PM; the middle pyramidal layer, P; the inner molecular layer, M. Notice the presence of the lateral ventricle (lV). H&E/scale bar 50.

The examination of the H&E stained sections of the PTZ group rats revealed an apparent decrease in the pyramidal cell number in area CA1 of the hippocampus proper. This neural loss was more evident in area CA3 of the hippocampus proper than in the control group. The administration of CoQ10 showed an improvement of their histological structure greater than that of the PTZ group. The pyramidal cell layer of CA3 demonstrated an increased pyramidal cell number in the VPA + CoQ10 group compared to those of the PTZ/VPA group. Most of the pyramidal cells exhibited large rounded vesicular nuclei. A few degenerated neurons with deeply stained cytoplasm and per-neuronal spaces were also encountered in the CA3 area (Figure 6).

**Figure 7:**
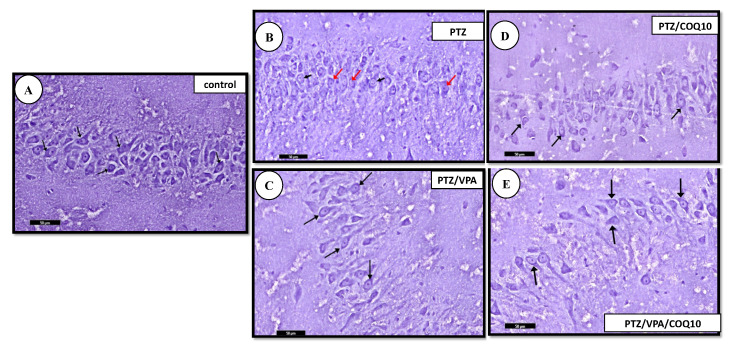
Photomicrographs of the Toluidine blue-stained section of the hippocampus of all of the rat groups. (**A**) Control group showing that the cytoplasm of the pyramidal cells appears heavily studded with Nissl granules (↑). (**B**) PTZ group showing an apparent decrease in the Nissl granule content (red ↑) of the pyramidal cells of the pyramidal layer, compared to that of group I (control, non-epileptic rats). The pyramidal layer with few of the pyramidal cells has large vesicular nuclei (black ↑). (**C**) PTZ/VPA group showing an apparent increase in the Nissl granule content (↑). (**D**) PTZ/COQ10 group showing an apparent moderate increase in the Nissl granule content (↑) of the pyramidal cells of the pyramidal layer. (**E**) PTZ/VPA/COQ10 group showing an apparent increase in the Nissl granule content (↑). Toluidine blue; scale bar 50 µm.

Nissl-stained sections: The PTZ group showed an apparent decrease in the Nissl granule content of some of the pyramidal cells of areas CA2 and CA3 of the hippocampus proper compared to group I. The administration of CoQ10 showed an apparent increase in the Nissl granule content of most of the pyramidal cells of area CA3 processes, compared to that of the PTZ group, and then an apparent moderate increase in the Nissl granule content of most of the pyramidal cells of the area CA3 processes in the PTZ + VPA + CoQ10 group compared to that of the PTZ/VPA group (Figure 7).

**Figure 8:**
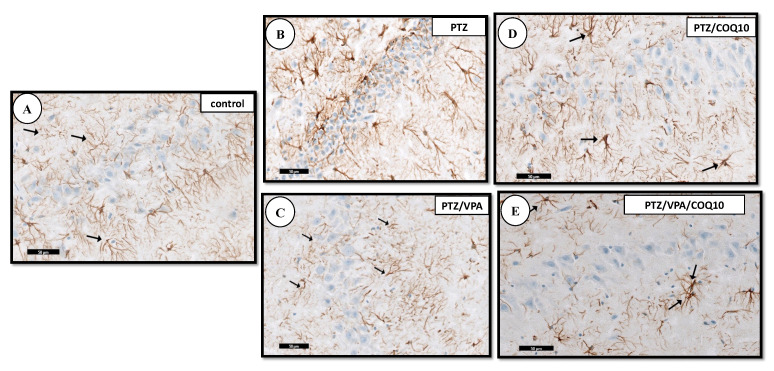
A photomicrograph of the CA3 region of the hippocampus of all of the rat group. (A) Control group showing the positive reaction for GFAP immuno-staining in the cytoplasm of astrocytes (↑). (**B**) PTZ showing an apparent increase of the positive brownish reaction for GFAP immuno-staining in the cytoplasm of astrocytes, compared to that of group I (the negative control, non-epileptic rats). (**C**) PTZ/VPA showing an apparent decrease of the positive brownish reaction for GFAP immuno-staining in the cytoplasm of the astrocytes. Notice that the astrocytes present shorter processes (↑) compared to those of group II (the epileptic model group). (**D**) PTZ/COQ10 showing the apparent moderate decrease of the positive brownish reaction for GFAP immuno-staining in the cytoplasm of the astrocytes. (**E**) PTZ/VPA/COQ10 group showing the apparent decrease of the positive brownish reaction for GFAP immuno-staining in the cytoplasm of the astrocytes.

The immunohistochemical staining for the demonstration of GFAP within the astrocytes of the PTZ group showed an apparent increase in the number of GFAP-positive astrocytes, which appeared larger with longer-branched cytoplasmic processes. On the other hand, the administration of CoQ10 demonstrated an apparent moderate decrease in the number of the astrocytes in area CA3 compared to that of the astrocytes which were small with a few short-branched processes in the PTZ + VPA + CoQ10 group, as compared to that of the PTZ/VPA group (Figure 8).

##### Liver Histology

**Figure 9:**
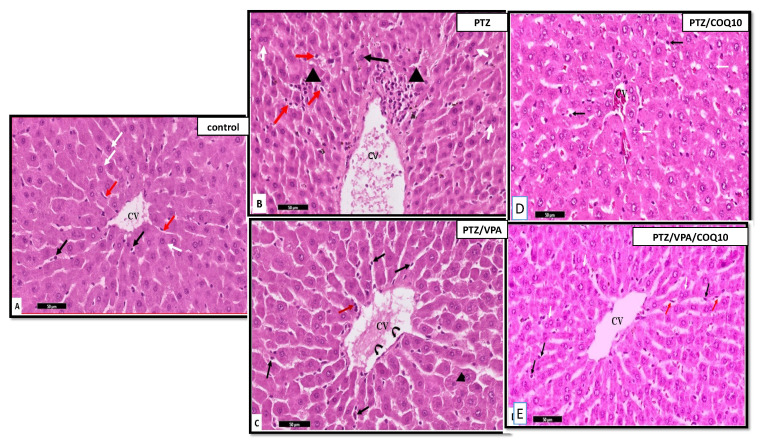
Photomicrographs of the rat liver section at the peri-central vein area of all of the rat groups. (**A**) Control group showing the polyhedral hepatocytes arranged radially in cords around the central vein (CV), with normal vesicular basophilic nuclei (white arrow). Notice the thin flat endothelial lining of the blood sinusoids (red arrows) and Von kupffer cells (black arrow). (**B**) PTZ group showing the hepatocytes with normal vesicular nuclei (arrow); some of them have a deep acidophilic cytoplasm. The binucleated hepatocytes are shown with white arrows. Notice the focal aggregation of mononuclear inflammatory cells between the hepatocytes with deep acidophilic cytoplasm and small, shrunken, deeply stained nuclei (Head arrow), and the sparse presence of the hypertrophied intra-sinusoidal cells (Von Kupffer cells) (red arrow) in-between the hepatocytes. (**C**) PTZ/VPA group showing the hypertrophied Von Kupffer cells (→) in-between the hepatocytes, which are surrounded by flat and low cubical cells (curved arrow). Notice the micro-vacuolation of the cytoplasm of some of the hepatocytes with small pyknotic nuclei (Head arrow), and the mononuclear cellular infiltration in-between the hepatocytes (red arrow). (**D**) PT Z/COQ10 showing the hepatocytes with normal architecture near to the control. (**E**) PTZ/VPA/COQ10 showing the polyhedral hepatocytes with normal vesicular basophilic nuclei and granular cytoplasm (white arrow). Notice that a few hepatocytes appeared with slightly vacuolated cytoplasm, especially in the perinuclear region (black arrows), the thin endothelial lining of the blood sinusoids (red arrows), and (**E**). H&E stain-group/scale bar ((**A**–**I**) 50 µm).

**Figure 10:**
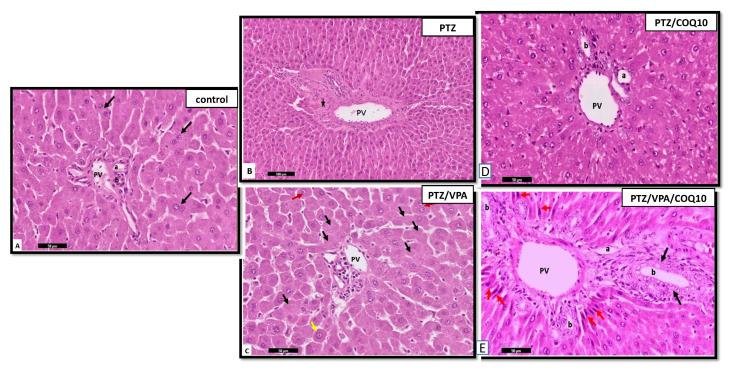
Photomicrographs of the rat liver sections at the portal area of all of the rats groups. Control group showing the portal vein (PV), Bile ducts (**b**) and hepatic artery (**a**). Some are binucleated (black arrow) (**A**). PTZ group showing the dilated portal vein (PV). Notice the heavy cellular infiltration (*) (**B**). (**C**) PTZ/VPA group showing a few normally arranged hepatocytes (red arrow); some of them show the loss and fading of nuclei (karyolysis) (Black arrow) and megalocytes (yellow arrow). (**D**) PT Z/COQ10 showing the hepatocytes with normal architecture near to the control. (**E**) PTZ/VPA/COQ10 showing the proliferating bile duct (black arrow) and the presence of newly formed hepatocytic cells with relatively rounded to elongated, large, dark nuclei (red arrow) (**E**). H&E stain/scale bar ((**A**–**I**) 50 µm).

The examination of the H&E of the liver sections in the PTZ group showed marked inflammatory cellular infiltration around the central veins and near the portal tracts. This finding was worse in the stained histological sections of the livers of the rats that received PTZ/VPA, which revealed that both stroma and parenchyma were greatly affected. However, the administration of CoQ10 showed amelioration in the previously observed lesions in the liver of the PTZ+CoQ10 group in comparison with PTZ alone. Furthermore, the PTZ + VPA + CoQ10 group showed a marked regression of the degenerative and inflammatory changes compared to those of the PTZ/VPA group, which received only VPA (Figure 9 and Figure 10).

**Figure 11:**
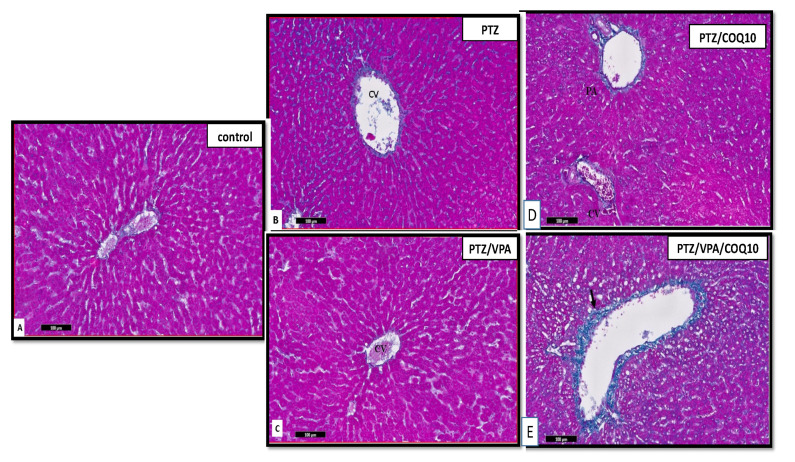
Photomicrographs of the stained liver sections (portal vein) of all of the rat groups. (**A**) Control group showing fine, blue-stained fibers between the radially arranged hepatocytes around the central vein and around the portal area (P). (**B**) PTZ group showing condensed, green-stained fibers between the radially arranged hepatocytes around the central vein (CV) and around the portal area (P). (**C**) PTZ/VPA group showing the apparent moderate increase in the green collagenous fibers. (**D**) PTZ/COQ10 showing the apparent decrease in the green, collagenous fibers around the central vein (CV) and portal areas (PV). (**E**) PTZ/VPA/COQ10 group showing fine, green, collagenous connective tissue between the cords of the hepatocytes, around the central vein and the walls of large vessels. (→). Masson trichrome stain/scale bar ((**A**–**E**) 100 µm).

The sections stained with Masson trichrome stain in the PTZ group showed increased collagen deposition around the portal areas and central veins. In addition, there was thick collagen fiber deposition surrounding the hepatic lobules enclosing the hepatic nodules. This finding was worse in the stained histological sections of the liver of the PTZ/VPA group. There was an apparent moderated increase in the collagen fibers in the stroma. The increase in collagen fibers was detected around the central veins surrounding the structure of the portal area. The administration of CoQ10 showed an apparent slight decrease in the collagen content in the stroma, which was observed in the PTZ+CoQ10 group. The collagen fibers were detected around the central veins and surrounding the portal areas’ structures, as compared to the PTZ group (Figure 11).

The PTZ+VPA+CoQ10 group showed delicate collagen fiber deposition extending between the portal areas which was much less than that observed in the PTZ/VPA group. In addition, few collagen fibers were seen surrounding the central veins, as in the control group. However, thin collagen fiber deposits could be detected between the hepatocytes and the portal area (Figure 10).

## 4. Discussion

This study’s PTZ injection-induced convulsion and significant increase in the average Racine score are consistent with Schmoll [18], who reported that the kindling criterion was induced by two doses of PTZ given 25 days apart. Treatment with VPA and CoQ10 decreased the Racine score significantly. Sefil et al. [19] reported that VPA completely prevented generalized seizures. VPA reduced the glutamate and aspartate release in the brain of the PTZ-kindled rats [20]. This explains our results, where there was a significant decrease of the glutamate concentration in the hippocampus tissue in the PTZ/VPA group compared to the PTZ rats. The PTZ/VPA/CoQ10 rats showed a significantly decreased Racine score from the second week to the end of the eighth week compared to the PTZ/VPA group. Sattarinezhad et al. [21] concluded that the administration of CoQ10 attenuated the seizures induced by PTZ.

CoQ10 exhibited a significant delay to the development of PTZ kindling and a significant delay in the latency to the onset of GTCS compared with the VPA group. In our study, CoQ10 reduced the duration of the GTCS induced by PTZ injection and decreased the mortality rate in the PTZ/VPA and PTZ/CoQ10 rats. Tawfik [22] reported that CoQ10 is considered to be a safe and effective adjuvant to phenytoin therapy in pilocarpine-induced seizures.

The PTZ/COQ10 and PTZ/VPA/CoQ10 rats exhibited significantly high delay ranges on the rotarod. In addition, Bath et al. [23] found several effects of VPA treatment on the rotarod task which were indicative of impairments in both essential motor coordination and balance.

The PTZ and PTZ/VPA rats showed a significant increase in the ROS concentration in their hippocampal tissues. In addition, it was previously demonstrated that valproic acid could be responsible for increased oxidative stress and lipid peroxidation with long-term use, possibly through metabolism to reactive epoxide intermediates [24]. Therefore, it is proposed that neuroprotective antioxidants could be applied as an add-on therapy for epileptic patients, leading to reduced neurodegeneration and epileptogenesis [25].

Interestingly, in our study, CoQ10 significantly downregulated the MDA, PC, and ROS levels, and up-regulated the GSH levels in the hippocampus tissue of epileptic rats compared to PTZ and PTZ/VPA rats. CoQ10 reduces the systemic oxidative stress response and enhances the antioxidant capacity in epileptic rats. Sakhaie et al. [26], Nagib et al. [27], and Bhardwaj and Kumar [28] stated that CoQ10 significantly reversed the oxidative damage.

The PTZ group showed a significant increase in the glutamate concentration in their hippocampus tissue. Furthermore, all of the treated rats showed a significantly reduced glutamate concentration compared with the PTZ group. Tawfik [22] found that CoQ10 decreased the glutamate concentration.

Neurodegenerative alterations were found in the pyramidal cells of the CA3 region of the hippocampus proper in the PTZ group, confirming prior findings. The PTZ/VPA rats’ hippocampus-appropriate slices revealed an enhanced histological structure. Our findings were consistent with Sedky et al. [29], who reported that cytopathological changes are characterized by neuronal shrinkage with a dark cytoplasm, and pyknotic nuclei in the pyramidal neurons of the hippocampus of the PTZ-kindled rats. They reported that the PTZ/VPA rats exhibited a significant decrease in oxidative stress and amelioration in the pathological changes caused by PTZ. The proper hippocampus sections of the PTZ/CoQ10 and PTZ/VPA/CoQ10 groups showed improved histological structures compared to those of the PTZ group and VPA group. Nagib et al. [27] reported a potential neuroprotective effect of CoQ10 in PTZ-induced seizures.

A decrease in Nissl granule content was detected in the hippocampus neurons in the epileptic rats. It has been reported that CoQ10 can increase the proliferation of neural precursor cells in vitro. This might explain the increased content of Nissl granules in most of the pyramidal cells of area CA3, which were detected by the Nissl stain examination of the PTZ/CoQ10 and PTZ/VPA/CoQ10 groups, as compared to that of the PTZ/VPA rats.

An increase in the GFAP-positive astrocytes was detected in area CA3 in the epileptic rats. Conversely, the immunohistochemical stain for GFAP demonstrated an apparent decrease in the astrocytes in area CA3 in the PTZ/VPA/CoQ10 rats. Our findings are in accordance with Nagib et al. [27].

The PTZ group exhibited a significant increase in TNF-α, which is in line with previous studies [30,31], while CoQ10 exhibited a significant decrease in TNF-α. In addition, the PTZ/VPA/CoQ10 group showed a significant decrease in TNF-α, in contrast with the VPA group. It has been demonstrated that CoQ10 can exert anti-inflammatory effects by inactivating NF-κB, which is responsible for the overexpression of pro-inflammatory cytokines [32].

Liver enzymes were decreased in the CoQ10-treated rats. Previous studies reported that CoQ10 protects the liver from acute acetaminophen-induced hepatotoxicity [33]. The liver of the PTZ/VPA rats showed a significant decrease in antioxidant GSH and a significant elevation in protein oxidation (PC) and lipid peroxidation (MDA) compared to the PTZ-kindled rats. Similar findings were observed by Guna et al. [34]. This could be explained by Schulz et al. [35], who stated that the decreased GSH level in the PTZ rats indicates free radical production, and that GSH was depleted during defense against oxidative stress. Abdel-Dayem et al. [36] demonstrated that VPA evoked a threefold rise in MDA levels and a 35% reduction in the levels of reduced GSH. The VAP group exhibited a significant increase in their liver ROS concentration compared with the PTZ rats. In contrast, the PTZ/CoQ10 and PTZ/VPA/CoQ10 groups showed a significant increase in GSH and a significant decrease in MDA and PC compared to the PTZ and PTZ/VPA rats. Similar results were observed by Esfahani et al. [37], who reported that CoQ10 showed a hepatoprotective effect in a thioacetamide-induced liver damage model. Emam et al. [38], found that CoQ10 resulted in a significant reduction in liver MDA and restored liver GSH in lipopolysaccharide-induced liver injury.

CYP2E1-induced toxic metabolites coupled with oxidative stress from ROS are proposed to be essential mediators of liver injury by the promotion of inflammatory and fibrogenic pathways to facilitate the recruitment of leukocytes and the activation of hepatic stellate cells (HSCs) [39]. This could confirm the histological results, such as the apparent increase in collagen fibers shown by the Masson trichrome stain in the PTZ and PTZ/VPA rats. This coincided with Sedky et al. [29], who mentioned that the PTZ and PTZ/VPA groups exhibited an apparent increase in collagen fibers around the central vein, portal area, and extending in-between the hepatocytes. Mantle and Hargreaves [40] reported that CoQ10 prevented liver injury by decreasing CYP2E1. The collagen contents of the PTZ/CoQ10 and PTZ/VPV/CoQ10 rats were comparable with the control rats. CoQ10 significantly decreased the liver collagen content stained with Sirus red stain [41].

The liver cells exhibited macro-vesicular and micro-vesicular cytoplasmic vacuoles, deep acidophilic cytoplasm, and small pyknotic nuclei in the PTZ/VPA groups, which appeared with apoptotic figures. Micro-vesicular hepatosteatosis is a typical feature of VPA toxicity, and is suggestive of mitochondrial involvement, especially involving β-oxidation [42]. Hypertrophied Von-Kupffer cells were increased in the PTZ and PTZ/VPA rats. Kupffer cells are reemerging as a critical mediator of liver injury. Ahmed et al. [43] stated that hepatocyte damage initiates hepatic fibrosis, leading to Kupffer cells’ activation and subsequent cytokine release. These factors activate HSCs, which proliferate and transform into myofibroblast-like cells that deposit large amounts of connective tissue components.

## 5. Conclusions

In the current work, the administration of CoQ10 adjacently with valproic acid in epileptic rats can produce a potential therapeutic effect in the reduction of the hepatotoxicity induced by VPA.

## Figures and Tables

**Table 1:** Scoring system for seizure activity (Racine 1972).

Stage 0	No Response
Stage 1	Hyperactivity, vibrissae twitching
Stage 2	Head nodding, head clones and cyclonic jerk
Stage 3	Unilateral forelimb clones
Stage 4	Rearing with bilateral forelimb clones
Stage 5	Generalized tonic–clonic seizure (GTCS) with loss of righting reflex.

**Table 2:** Effect of CoQ10on antioxidants and oxidative stress markers in the hippocampus of rats subjected to PTZ kindling.

Rats Groups *N* = 6	Antioxidants and Oxidative Stress Markers			
	GSH ng/mg	ROS Unit/mg	MDA nmol/mg	PC nmol/mg
Control	101.18 ± 22.07	1077.18 ± 41.16	1.97 ± 0.41	1.216 ± 0.1
PTZ	19.5 ± 4.7 S *	1271.2 ± 48.07 *	7.64 ± 0.6 *	1.945 ± 0.12 *
PTZ/VPA	85.34 ± 6.5 ^#,^	1236.6 ± 41.53	6.70 ± 0.7 ^#^	1.737 ± 0.09 ^#^
PTZ/COQ10	95.88 ± 2.45 ^#^ ↑80%	1127.5 ± 41 ↓11%	3.52 ± 0.66 ^#^ ↓50%	1.422 ± 0.16 ^#^
PTZ/VPA + COQ10	113.38 ± 3.5 ^#,$^ ↑25%	1062.64 ± 40.13 S ^#,$^ ↓14%	3.37 ± 0.46 ^#,$^ ↓54%	1.412 ± 0.13 ^#^

Data are expressed as the mean ± the standard error of the mean, one-way ANOVA, and post hoc Tukey’s test. COQ10: Coenzyme Q10; GSH: reduced Glutathione; MDA: Malondialdehyde; PC: Protein Carbonyl; PTZ: Pentylenetetrazole; ROS: reactive oxygen species; VPA: Valproic acid. * *p* < 0.05 compared to the control; # *p* < 0.05, compared to the PTZ group alone; $ *p* < 0.05 for the PTZ/VPA + COQ10 group compared to the PTZ/VPA group. Percentages were calculated in comparison to the PTZ group ↑ = Increase, ↓ = Decrease. *n* = number.

**Table 3:** Effect of CoQ10 on antioxidants and oxidative stress markers in the hepatic tissue of rats subjected to PTZ kindling.

Rats Groups *N* = 6	Antioxidants and Oxidative Stress Markers				
	GSH ng/mg	ROS Unit/mg	MDA nmol/mg	PC nmol/mg	CYP2EL ng/mg
Control	135.42 ± 4.08	1077.20 ± 50.4	2.35 ± 0.4	1.564 ± 0.09	18 ± 2.053
PTZ	30.56 ± 4.41 *	1395.5 ± 58.7 *	9.74 ± 0. 3 *	2.230 ± 0.09 *	96.42 ± 4.71 *
PTZ/VPA	95.12 ± 5.23 ^#^	1204 ± 32.4	9.34 ± 0.35	1.95 ± 0.06	73.44 ± 5.78 ^#^
PTZ/COQ10	130.3 ± 6.5 ^#^ ↑76%	1116.9 ± 12.6 ^#^ ↓20%	3.17 ± 0.7 ^#^ ↓67%	1.852 ± 0.08 ^#^ ↓17%	61.1 ± 1.055 ^#^ ↓36%
PTZ/VPA + COQ10	133.7 ± 3.3 ^#,$^ ↑29%	998.1 ± 32 ^#,$^ ↓25%	5.22 ± 0.61 ^#,$^ ↓44%	1.82 ± 0.04 ^#^	67.04 ± 4.097 ^#^

Data are expressed as the mean ± the standard error of the mean, one-way ANOVA, and post hoc Tukey’s test. COQ10: Coenzyme Q10; GSH: reduced Glutathione; MDA: Malondialdehyde; PC: Protein Carbonyl; PTZ: Pentylenetetrazole; ROS: reactive oxygen species; VPA: Valproic acid.* *p* < 0.05 compared to the control; # *p* < 0.05, compared to the PTZ group alone; $ *p* < 0.05 for the PTZ/VPA + COQ10 group compared to the PTZ/VPA group. The percentages were calculated in comparison to the PTZ group. ↑ = Increase, ↓ = Decrease. *n* = number.

## Data Availability

The data are contained within the article.
